# Seroprevalence of Hepatitis B Virus and Associated Factors among Pregnant Women Attending Antenatal Care Services at Public Health Facilities in Nekemte Town

**DOI:** 10.1155/2021/9572235

**Published:** 2021-12-27

**Authors:** Sinkinesh Eba, Gemechu Kejela, Afework Tamiru

**Affiliations:** ^1^Department of Medical Laboratory, Nekemte Specialized Hospital, Nekemte, Ethiopia; ^2^Department of Public Health, Institute of Health Sciences, Wollega University, Nekemte, Ethiopia

## Abstract

**Background:**

Viral hepatitis is an emerging global health problem. A pregnant mother infected with the hepatitis B virus has a high rate of vertical transmission, causing adverse fetal and neonatal outcomes. Understanding the magnitude of the problem and associated factors has paramount importance to avert such adverse fetal and neonatal outcomes. Therefore, the main aim of this study was to assess the seroprevalence of hepatitis B virus and associated factors among pregnant women attending antenatal care clinics at public health facilities in Nekemte town.

**Methods:**

An institutional-based cross-sectional study was conducted among 277 pregnant women attending antenatal care at public health facilities in Nekemte town from June 1 to July 30, 2020. All public health institutions in Nekemte town (two hospitals and one health center) were recruited, and study participants were selected by using a systematic sampling method. The data were collected using pretested and structured questionnaires using a face-to-face interview, and a blood sample was collected to test for hepatitis B surface antigen. Logistic regression analysis was employed to identify factors significantly associated with hepatitis B virus infection. Variables with a *p* value < 0.05 were considered statistically significant predictors of the outcome variable.

**Result:**

The overall seroprevalence of hepatitis B virus infection was 16 (5.8%) [95% CI: 3.2-8.7], which indicates intermediate endemicity. History of abortion (AOR =6.155; 95% CI: 1.780, 21.291), history of contact with hepatitis patient (AOR =7.178; 95% CI: 1.702, 30.279), and having multiple sexual partners (AOR =6.788; 95% CI: 1.701, 27.086) had a statistically significant association with hepatitis B surface antigen seropositivity.

**Conclusion:**

Hepatitis B virus seroprevalence among pregnant women in this study shows intermediate endemicity. Therefore, health professionals should provide health information on the risk of having multiple sexual partners, risk factors of unprotected contact with hepatitis patients, and abortion for pregnant women during their antenatal care visits.

## 1. Background

Hepatitis B virus (HBV) is a major blood-borne and sexually transmitted infectious agent that is a significant global public health issue [1]. It is the most common cause of serious viral hepatitis, leading to acute and chronic infections of the liver. Chronic hepatitis can cause cirrhosis and hepatocellular carcinoma [2, 3]. Hepatitis B surface antigen (HBsAg) is the serologic hallmark of HBV infection that establishes its diagnosis [4]. Due to largely its asymptomatic nature, viral hepatitis is a silent epidemic. As a result, most people are unaware of their infection. Hepatitis B virus is one of the vaccine-preventable diseases that have a safe and effective vaccine [5].

Hepatitis B virus is extremely infectious (about 100 times as infectious as HIV) [6]. Worldwide, more than 300 million people have chronic liver infections, and about 600,000 people die annually from acute or chronic complications of hepatitis B infection [7]. The prevalence of chronic HBV infection is highest in Sub-Saharan Africa and East Asia among the adult population, which was 5 and 10 percent, respectively [3], whereas in the Western Pacific region and the African region, it was 6.2 and 6.1 percent, respectively [8]. In Ethiopia, the study shows that there are 7.4 percent among the population and 4.7 percent among pregnant women [9].

Hepatitis B virus can be transmitted from mother to child during pregnancy, during delivery, and after delivery through breast feeding [9, 10]. The virus is most commonly transmitted from mother to child during birth [3, 9]. About 80%-90% of infants infected during the first year of life and 30%-50% of children infected before the age of 6 years develop chronic HBV infection. Additionally, 15-25% of adults who become chronically infected during childhood go on to develop hepatitis B-related liver cancer/cirrhosis [2]. Accordingly, chronicity is much more expected among individuals infected during infancy and childhood, compared to individuals infected during adulthood [11].

Vertical transmission remains the most frequent route of infection, particularly in endemic areas where up to 20% of women of childbearing age may have HBV and approximately one-third of these are responsible for chronic HBV infection [12]. Hepatitis B infection is associated with death arising from cirrhosis, liver, and nonliver cancers. Transmission of HBV infection also resulted from exposure to contaminated blood or body fluids, unprotected sexual contact with an infected person, blood transfusion, and use of contaminated needles, syringes, and other sharp materials [13]. Also, tattooing for cosmetics, ear piercing, and tonsillectomy are associated with HBV infection [10].

The World Health Organization (WHO) recommends that pregnant women attending antenatal care (ANC) should be routinely screened for HBV and administered immune globulin and vaccine within 12 hours of birth for infants whose mothers are tested positive for the infection. Even though the program is crucial in minimizing the risk of HBV among newborns, the major part of HBV spread is also left with contact networks of the infected mothers [14].

Preventing mother-to-child transmission of HBV is important for reducing the burden of the disease in Sub-Saharan Africa, where the problem is endemic. An effective strategy for reducing the incidence of chronic infections is maternal screening combined with postexposure prophylaxis consisting of HBV vaccination immediately after delivery in all infants born to HBsAg-positive mothers, jointly with immunoglobulin prophylaxis [15]. In Ethiopia, a screening program for pregnant mothers is being implemented in public health facilities at antenatal care points. Moreover, universal immunization of infants against HBV is performed as part of the National Expanded Program on Immunization starting at six weeks [16–18].

Despite its effects on both the mother and child, the routine screening of pregnant women for HBV is not practiced in many Ethiopian public health facilities [19]. Also, the program is fragmented, not running regularly, and postexposure prophylaxis is lacking [17]. The absence of a regular HBV screening program could be partly explained by the lack of awareness on the overall burden and associated factors of hepatitis B among pregnant women in Ethiopia by health professionals and policymakers [18]. Therefore, the main aim of this study was to assess the seroprevalence of hepatitis B virus and associated factors among pregnant women attending antenatal care clinics at public health facilities in Nekemte town.

## 2. Methods

### 2.1. Study Area and Period

A health facility-based quantitative cross-sectional study was conducted in Nekemte town public health facilities from June 1 to July 30, 2020. Nekemte town is found in East Wollega zone, Oromia regional state, west of Ethiopia, at a distance of 328 kilometers from Addis Ababa. The catchment population getting services from Nekemte town health facilities are about 1,902,380 (male 998,333 and female 904,047). Nekemte public health facilities include Nekemte Specialized Hospital (NSH), Wollega University Referral and Teaching Hospital (WURH), and Nekemte Health Center. Both hospitals are tertiary level hospitals that provide health services for the population of the catchment area and have a different department that provides specialized services in outpatient, inpatient, and operation theatre departments. All health facilities provide care for pregnant mothers widely in ANC, intrapartum and postpartum periods. HBV screening is expected to be undergone by all pregnant mothers who had antenatal care follow-up since it is one of the most important routine investigations during their first ANC follow-up, but free vaccination is not started yet.

### 2.2. Population

All pregnant women visiting ANC clinics at public health facilities in Nekemte town were the target population. Pregnant women who visited ANC at public health facilities of Nekemte town during the study period and fulfilled the inclusion criteria were the study population.

### 2.3. Eligibility Criteria

Women who were having antenatal care follow-up at Nekemte Specialized Hospital, Wollega University Referral and Teaching Hospital, and Nekemte Health Center during the study period were included in the study. Women required urgent intervention and pregnant women who have been vaccinated for HBV were excluded from the study.

### 2.4. Sample Size Determination and Sampling Procedure

The sample size was calculated by using a single population proportion formula [20], considering the following assumptions: prevalence of *p* = 6.3% taken from a previous study conducted in Harari city, Eastern Ethiopia [5], 95% CI, and 3% degree of precision, because when the prevalence of the disease is going to be below 10% or more than 90%, the degree of precision is taken as half of the prevalence [21]. From this, sample the size was calculated as follows:

(1) $$\begin{array}{c} n={\left(\frac{Z\alpha }{2}\right)}^{2}\frac{p\left(1-p\right)}{d^{2}},\\ n=\frac{{\left(1.96\right)}^{2}*0.063\left(1-0.063\right)}{0.0009},\\ n=\frac{3.8416*0.059031=252}{0.0009}. \end{array}$$

By adding 10% nonresponse rate, the final sample size becomes 277.

In Nekemte town, there are two public hospitals and one health center. All the three health institutions are included in the study. Then, proportional allocation for the calculated sample size was made based on a previous two-month average ANC follow-up from the registration book for all public health facilities that are used to recruit the actual sample. The sample was selected by using a systematic sampling method, after calculating the sampling interval (*K*). The interval was equal since the sample is proportionally allocated to all public health facilities. Then, the first pregnant mother was randomly selected by a lottery method.

### 2.5. Measurements

In this study, seroprevalence was defined as the proportion of pregnant women with hepatitis B surface antigen-positive status attending ANC at Nekemte public health institution during the study period. Seropositive is defined as the presence of hepatitis B surface antigen in the serum among pregnant women attending ANC at Nekemte public health institution during the study period. Seronegative is defined as the absence of hepatitis B surface antigen in the serum among pregnant women attending ANC at Nekemte public health facilities during the study period. Household contact with hepatitis patients was defined as the presence of unprotected contact with positive HBsAg in the family among pregnant women attending ANC during the study period. Unprotected contact is defined as a contact with HBV patients without taking appropriate precaution/protective measures, because a person may get hepatitis B virus if he/she had unprotected sex with someone who is infected with the virus; he/she had contact with blood, saliva, semen, or vaginal secretions of HBV patients; and he/she had shared needles and syringes with individuals infected with the virus.

### 2.6. Data Collection Tools and Procedures

To collect the data, the questionnaire was adopted from the WHO guidelines and similar literature. Data collection was implemented through a face-to-face interview by using a pretested structured questionnaire which consists of sociodemographic and economic characteristics, health care delivery system-related factors, traditional practices, and behavioral-related factors. A blood sample test was designed to collect the results of HBsAg from study participants by requesting a laboratory investigation paper. One-day training was given for four BSc midwifery nurses for data collection, four laboratory technicians for sample collection and testing blood for HBV, and two BSc nurse supervisors for both hospitals and health center.

### 2.7. Specimen Collection and Processing

Five milliliters of venous blood was drawn under aseptic conditions in disposable vacutainer tubes by trained laboratory personnel. These tubes were labeled with the participant's code. The blood was centrifuged at 3000 revolutions per minute (RPM) for at least 10 minutes at room temperature. Then, serum was separated by a pasture pipette carefully not to include cell parts. The rapid test was performed to deliver the result of the pregnant women at the time of screening. The leftover serum was separated and collected in Eppendorf tubes, stored at -20°C [10]. Finally, the positive result by a rapid test kit was confirmed by ELISA in Nekemte blood bank.

### 2.8. Data Quality Assurance

To ensure the quality of data, questionnaires developed in English were translated to a regional language (Afan Oromo) and again translated back to English by a bilingual expert. To make sure that the questionnaire is appropriate and understandable, it was pretested on 5% of pregnant women at Sire Primary Hospital before the actual data collection was conducted. The collected data were checked daily for consistency and accuracy. The training was given to supervisors and data collectors on data collection techniques. Standardized operational procedures were strictly followed during preanalysis, analysis, and postanalysis. To assure the quality and accuracy of the laboratory blood test result, standard operating procedures were strictly followed during blood sample collection, storage, and analytical process. Storage conditions and expiry date of reagents were checked. Positive and negative control sera were run following the manufacturer's recommendation of the kit. Finally, reliability was checked by using Cronbach's alpha (0.681).

### 2.9. Data Processing and Analysis

The collected data were checked for completeness and consistency, and it was coded and entered into epi data version 3.1 and exported to SPSS version 24.0 and cleaned for analysis. Frequency and summary statistics were used to describe the study population. For statistical significance, crude odds ratios (COR) with their 95% CI were estimated using binary logistic regression. Those independent variables with a *p* value < 0.20 at the bivariate level were included from the multivariable logistic regression model. The association between independent and dependent variables was assessed using an adjusted odds ratio (AOR) with a 95% confidence interval in multivariable logistic regression. Finally, variables with a *p* value < 0.05 were considered statistically significant predictors of the outcome variable.

## 3. Results

### 3.1. Sociodemographic Characteristics

A total of 277 pregnant mothers attending ANC services at public health facilities in Nekemte town were involved in the study, making a response rate of 100%. The age of the respondents ranged from 18 to 40 with a mean of 25.88 and standard deviation of (SD ± 4.782). One hundred nineteen (43%) of the respondents were within the age group of 18-24 years. The majority (237; 85.6%) of the respondents were urban dwellers. Regarding the ethnicity of the respondents, 228 (82.3%) were Oromo. Two-thirds of the respondents (181; 65.3%) were followers of the Protestant religion, followed by Orthodox (64; 23.1%) and Muslim (32; 11.6%). About two-fifths (106; 38.6%) of the respondents were not employed. The majority of the respondents were married (270; 97.5%). Only ten (3.6%) respondents had no formal education, and 121 (44%) of them had completed education at a college or university. More than half of the respondents (154; 55.6%) had a monthly income of greater than 1500 ETB which is equivalent to 35.7 dollars (Table 1).

### 3.2. Obstetric and Other Health Service-Related Factors

Almost all (265; 95.7%) of the respondents had no history of blood transfusion. Similarly, more than two-thirds (213; 76.9%) of the respondents had no history of hospital admission in their lifetime. About 215 (77.6%) of the study respondents had no history of abortion. Majority of the respondents had first pregnancy (114; 41.2), followed by second pregnancy (93; 33.6). The majority of them attended antenatal care in the second trimester (120; 43.3), and half of the participants had a history of previous delivery at health institutions (Table 2).

### 3.3. Traditional Practice and Behavioral-Related Factors

Among the study respondents, 62 (22.4%), 205 (74%), 260 (93.9%), and 20 (7.2%) of them had a history of body tattooing, history of female genital mutilation, history of ear piercing, and history of having multiple sexual partners, respectively **(**Table 3**)**.

### 3.4. Prevalence of HBV Infection

**Figure 1:**
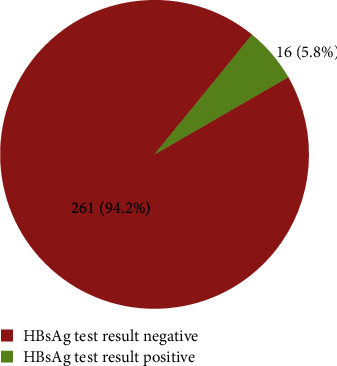
Prevalence of hepatitis B virus among pregnant women attending antenatal clinic at public health facilities in Nekemte town, Western Ethiopia, 2020.

From the 277 study participants, the prevalence of HBsAg was 16 (5.8%) [95% CI: 3.2-8.7]. Positive samples were repeated by ELISA, and the results were positive, indicating there were no discordant results. This shows no false positives in the rapid test kit (Figure 1).

### 3.5. Associated Factors for Hepatitis B Virus Infection

The binary logistic regression analysis was conducted to select candidate variables for multivariable logistic regression analysis. Variables with a *p* value less than 0.2 in binary logistic regression were selected for the final model. Multivariable logistic regression analysis was done to identify factors associated with hepatitis B infection among pregnant women attending ANC at public health facilities in Nekemte town. In the binary logistic regression analysis, age, educational status, place of residence, history of contact with hepatitis patients in the family, abortion, and history of multiple sexual partners had a significant association with hepatitis B infection.

In multivariable logistic regression analysis, history of contact with hepatitis patients in the family, abortion, and having a history of multiple sexual partners were associated with hepatitis B virus infection.

Pregnant women having a history of contact with hepatitis patients were 7.2 times (AOR = 7.178; 95% CI: 1.702, 30.279) more likely of being infected by HBV than pregnant women who had no history of having contact with a hepatitis patient in a household or family. The study also showed that participants who had a history of abortion were 6 times more likely to develop HBV infection than those who had no history of abortion (AOR = 6.155; 95% CI: 1.780, 21.291). The study revealed that women who had a history of multiple sexual partners were seven times more likely to be exposed to HBV than those women with no history of multiple sexual partners (AOR = 6.788; 95% CI: 1.701, 27.086) (Table 4).

## 4. Discussion

In this study, the overall seroprevalence of HBV positivity among pregnant mothers attending ANC services at public health facilities in Nekemte town was 16 (5.8%) [95% CI: 3.2-8.7]. According to the WHO classification, the prevalence of HBV infection in this study area can be categorized as an intermediate endemicity (2%-7%) [10].

The prevalence of this study is almost similar to the findings of the study conducted in Jimma (3.7%) [22], a study conducted in Debra-Tabor Hospital (5.3%) [23], a study conducted in Bahir Dar, northwest Ethiopia (4.7%) [24], a study conducted in Harari city (6.3%) [5], and a study conducted in Deder town, Eastern, Ethiopia (6.9%) [4]. This similarity may be as a result of similar sociodemographic and economic characteristics of the study population.

In contrary to this, the higher prevalence was reported from a study conducted in Gambella (7.9%) [7], a study conducted in Hawassa (7.8%) [25], a study conducted in Nigeria (19.5%) [26], and a study conducted in the Gambia (9.2%) [27]. The higher prevalence in the others studies may be since those studies are conducted in large cities where exposure to the disease is high. The present study is higher than studies conducted in developed nations like the USA (<2%) [14], Germany (0.48%) [28], Norway (0.1%) [29], and Switzerland (1.2%) [30]. The low prevalence in this study might be because of good screening of pregnant women for HBsAg and availability of vaccine in the aforementioned developed countries.

Generally, the variations in seroprevalence in Ethiopia and somewhere else might be because of differences in geographical regions, socioeconomic status, cultural and behavioral practices towards the risk of HBV infection, and health-related factors. Another reason for the difference might be due to the difference in awareness of the routes of transmission among the population, the difference in methodology of the studies, and sampling variability. It might also be due to the potential variability in sensitivity and specificity of the commercially available test kits used in each study.

The current study revealed that pregnant mothers who have a history of multiple sexual partners were seven times more likely to be infected with HBV compared to their counterparts. Previous studies also evidenced that acquisition of HBV infection is significantly higher among participants involved in multiple sexual practices [5, 7, 16]. This is because the history of having multiple sexual partners and unprotected sexual intercourse is closely related to STI, which can easily expose to HBV. This finding may be due to the reason that hepatitis B is a blood-borne virus; that blood, semen, and other body fluids are a common source of infection; and that sexual contacts serve as a mode of transmission. However, the finding is inconsistent with a previous study conducted in Bishoftu General Hospital, which stated that sexual intercourse would have less probability of transmission of HBV infection among the same study population [17].

In this study, pregnant mothers who experienced a history of abortion were 6 times more likely to be positive for HBsAg. Similar results were reported from a study conducted in Jimma [22], a study conducted in Arba Minch [18], a study conducted in Deder hospital [4], and a study conducted in Dessie, Ethiopia [31]. This might be attributed to poor practice of infection prevention control during abortion, contaminated instruments used during the procedure, and related activities that might increase the probability of acquiring HBV infection.

In this study, the odds of having hepatitis B virus infection among the study participants who had a history of contact with hepatitis patients in the family were 7.2 times higher than their counterparts. This finding is consistent with other studies that shows the risk of HBV transmission was higher among people in contact with chronically infected HBV subjects [23, 26, 32]. This might be due to unprotected contact during care for individuals and a lack of awareness in the mode of prevention.

## 5. Limitations of the Study

The study does not include private health facilities and a small sample size due to resource constraints and laboratory setup. There may be some biases to give accurate information when the interviewer asks about past exposures. Because of the expensive nature of the ELISA test, all study participants including negative and positive in rapid HBsAg test were not tested for confirmation by ELISA test. There was also social desirability bias due to some sensitive questions such as a report on sex partners.

## 6. Conclusions

The findings of this study showed that the magnitude of HBsAg seropositivity is 16 (5.8%) [95% CI: 3.2-8.7]. This shows almost medium endemicity of HBV infection according to the WHO criteria. Having a history of contact with hepatitis patients, having a history of multiple sexual partners, and having a history of abortion had a statistically significant association with the outcome variable.

As a result, screening of pregnant women for HBV regardless of the basis of risk factors and developing a strong prevention strategy targeting this group may reduce mother-to-child transmission of HBV infection. Special emphasis should be given to screening pregnant women who have risk factors like having contact with hepatitis patients, having multiple sexual partners, and having a history of abortion. Ethiopian government in general and the health authorities and health service providers in the study area in particular should increase HBsAg screening uptake for all pregnant women. Also, the Ethiopian government should avail vaccine for all women in the reproductive age group to reduce the prevalence of HBV among pregnant women. In this study, having history of multiple sexual partners is one of the risk factors for HBV. So, health professionals and other concerned bodies should create an awareness for women in the reproductive age group about the problems of having multiple sexual partners that can predispose them to HBV. Moreover, further large-scale studies should be done to ensure the independent forecasting of HBV infection.

## Figures and Tables

**Table 1:** Sociodemographic characteristics of pregnant women attending antenatal care at public health facilities in Nekemte town, Western Ethiopia, 2020 (*n* = 277).

Variables	Categories	Frequency	Percentage (%)
Age in year	18-24	119	43
	25-30	112	40.4
	≥31	46	16.6

Marital status	Single	7	2.5
	Married	270	97.5

Residence	Urban	237	85.6
	Rural	40	14.4

Ethnicity	Oromo	228	82.3
	Amhara	27	9.7
	Guraghe	22	7.9

Average monthly income	<500 ETB	14	5.0
	500-1500 ETB	109	39.4
	>1500 ETB	154	55.6

Occupation	Self employed	83	30
	Government employed	58	20.9
	Private employed	30	10.8
	Not employed	106	38.6

Educational status	No formal education	10	3.6
	Primary education	58	20.9
	Secondary education	88	31.8
	College and above	121	43.7

**Table 2:** Obstetric and other health service-related factors among pregnant women attending ANC at public health facilities in Nekemte town, Western Ethiopia, 2020.

Variables	Categories	Frequency	Percentage (%)
Number of pregnancies	First	114	41.1
	Second	93	33.6
	Three and above	70	25.3

Gestational age	First trimester	67	24.2
	Second trimester	120	43.3
	Third trimester	90	32.5

Place of delivery	Home	22	13.4
	Health institution	142	86.6

History of abortion	No	215	77.6
	Yes	62	22.4

History of blood transfusion	No	265	95.7
	Yes	12	4.3

History of hospital admission	No	213	76.9
	Yes	64	23.1

History of surgical procedure	No	244	88.1
	Yes	33	11.9

History of tooth extraction	No	183	66.1
	Yes	94	33.9

**Table 3:** Traditional practices and behavioral-related factors of HBV among pregnant women attending ANC services at public health facilities in Nekemte town, western Ethiopia, 2020.

Variables	Categories	Frequency	Percentage (%)
History of body tattooing	No	215	77.6
	Yes	62	22.4

History of genital mutilation	No	72	26
	Yes	205	74

History of ear piercing	No	17	6.1
	Yes	260	93.9

History of traditional tonsillectomy	No	249	89.9
	Yes	28	10.1

History of having multiple sexual partners	No	257	92.8
	Yes	20	7.2

History of contact with HBV patient	No	256	92.4
	Yes	21	7.6

**Table 4:** Multivariable logistic regression analysis of seroprevalence of HBV infection and associated factors among pregnant women attending ANC at public health facilities in Nekemte town, Western Ethiopia, 2020.

Variables	Category	HBsAg test result		COR (95% CI)	AOR (95% CI)
		Negative (*N*, %)	Positive (*N*, %)		
Age	18-24	11 (93.3)	8 (6.7)	0.757 (0.216, 2.646)	1.000 (0.211, 4.737)
	25-30	108 (96.4)	4 (3.6)	0.389 (0.093, 1.627)	0.286 (0.048, 1.724)
	≥31	42 (91.3)	4 (8.7)	1	1

Level of education	No formal education	8 (80.0)	2 (20.0)	5.800 (0.969, 34.72)	1.498 (0.166, 13.50)
	Primary school	53 (91.4)	5 (8.6)	2.189 (0.608, 7.884)	2.211 (0.40, 12.147)
	Secondary school	84 (95.5)	4 (4.5)	1.105 (0.288, 4.238)	1.272 (0.245, 6.589)
	College and above/university	116 (95.9)	5 (4.1)	1	1

Residence	Urban	226 (95.4)	11 (4.6)	1	1
	Rural	35 (87.5)	5 (12.5)	2.935 (0.962, 8.955)	2.022 (0.504, 8.122)

Income	<500	13 (92.9)	1 (7.1)	1.90 (0.212, 16.981)	1.04 (0.060, 18.174)
	500-1500	100 (91.7)	9 (8.3)	2.220 (0.766, 6.431)	1.605 (0.431, 5.974)
	≥1500	148 (96.1)	6 (3.9)	1	1

History of contact with HBV patient	No	245 (95.7)	11 (4.3)	1	1
	Yes	16 (76.2)	5 (23.8)	6.96 (2.156, 22.468)	7.178 (1.702, 30.27)^∗^

History of abortion	No	208 (96.7)	7 (3.3)	1	1
	Yes	53 (85.5)	9 (14.5)	5.05 (1.796, 14.172)	6.155 (1.78, 21.29)^∗^

History of having multiple sexual partners	No	247 (96.1)	10 (3.9)	1	1
	Yes	14 (70.0)	6 (30.0)	10.586 (3.36, 33.31)	6.79 (1.70, 27.086)^∗^

^∗^Statistically significant at *p* value < 0.05. ^1^Reference category.

## Data Availability

The data sets used and analyzed for the current study are available from the corresponding author on reasonable request.

## Abbreviations

ANC: Antenatal care
AOR: Adjusted odds ratio
CI: Confidence interval
COR: Crude odds ratio
ELISA: Enzyme-linked immunosorbent assay
HBsAg: Hepatitis B surface antigen
HBV: Hepatitis B virus
HIV: Human immunodeficiency virus
NSH: Nekemte Specialized Hospital
OR: Odds ratio
SD: Standard Deviation
SPSS: Statistical Packages for Social Science
STI: Sexually transmitted infections
WURH: Wollega University Referral Hospital.

## Supplementary Materials

Supplementary Materials The supplementary file contains a questionnaire prepared to assess seroprevalence of hepatitis B virus and associated factors among pregnant women attending ANC services at public health facilities of Nekemte town.
