# Supplementary material for: Seroprevalence of Hepatitis B Virus and Associated Factors among Pregnant Women Attending Antenatal Care Services at Public Health Facilities in Nekemte Town
Source: Int J Reprod Med. 2021 Dec 27;2021:9572235. doi: 10.1155/2021/9572235 (PMC8723869; doi:10.1155/2021/9572235)
Supplement: Supplementary Materials — The supplementary file contains a questionnaire prepared to assess seroprevalence of hepatitis B virus and associated factors among pregnant women attending ANC services at public health facilities of Nekemte town. [file 9572235.f1.docx]

| **Questionnaire in English version**  A questionnaire prepared to assess sero-prevalence of Hepatitis B Virus and Associated factors among pregnant women attending ANC services at public health facilities of Nekemte town.  Interviewer Name Questionnaire Number Date of interview  Participant Code Name of the health institution___ pregnant women registration number Categories Code ---------------  **Direction;** circle on response categories | | | | |
| --- | --- | --- | --- | --- |
| **S No** | **Questions** | | **Response** | **Remark** |
| **Part I ;- Socio-demographic and economic information** | | | | |
| 101 | Age (in years) |  | |  |
| 102 | Marital status? | 1. Single 2. Married 3. Widowed 4. Divorced | |  |
| 103 | Education Level? | 1. No formal education at all 2. Primary education 3. Secondary education 4. College level & above | |  |
| 104 | What is your occupational  status? | 1. Self employed 2. Government employed 3. Private employed 4. Not employed | |  |
| 105 | Number of pregnancy or gravidity | 1. First 2. Second 3. Third& above | |  |
| 106 | Average monthly income |  | |  |
| 107 | Where do you live? | 1. Urban 2. Rural | |  |
| 108 | What is your religion? | 1. Orthodox 2. Muslim 3. Protestant 4. Other specify ____________ | |  |
| 109 | Ethnicity | 1. Oromo 2. Amahara 3. Gurage 4. Tigre 5. Others, specify _____________ | |  |
| 110 | What is your gestational age? | 1. 1st trimester 2. 2nd trimester 3. 3rd trimester | |  |
| 111 | Place of previous birth | 1. no birth 2. home 3. health institution | |  |
| 112 | Have you ever had history of house hold contact? | 1. yes 2. No | |  |
| **Part II: - Questions towards health service related associated factors of**  **Hepatitis B infections.** | | | | |
| **S. No** | **Questions** | **Response** | | **Remark** |
| 201 | Is there any history of blood?  Transfusion | 1. Yes 2. No | |  |
| 202 | Have you ever admitted in hospital? | 1. Yes 2. No | |  |
| 203 | Do you expose to any surgical procedure? | 1. Yes 2. No | |  |
| 204 | Have you ever had history of tooth extraction? | 1. Yes 2. No | |  |
| 205 | Have you ever had history of abortion? | 1. Yes 2. No | |  |
| **Part III: - Questions towards traditional practice and behavioral related factors associated with hepatitis B infections** | | | | |
| 301 | Have you had a tattoo done in your life? | 1. Yes 2. No | |  |
| 302 | Is t h e r e a n y g e n i t a l Mutilation? | 1. Yes 2. No | |  |
| 303 | Is there any history of ear piercing | 1. Yes 2. No | |  |
| 304 | Have you ever had traditional tonsillectomy? | 1. Yes 2. No | |  |
| 305 | Did you have multiple sexual partner in  your life? | 1. Yes 2. No | |  |
| **Part IV: - Laboratory finding** | | | | |
| 401 | Serology (Laboratory findings) | 1. Positive 2. Negative | |  |

This is the end of the questionnaire. Thank you very much for taking time to answer these questions. We appreciate your help.
